# Antimicrobial Drug Consumption in Companion Animals

**DOI:** 10.3201/eid1102.040827

**Published:** 2005-02

**Authors:** Ole E. Heuer, Vibeke Frøkjær Jensen, Anette M. Hammerum

**Affiliations:** * Danish Institute for Food and Veterinary Research, Søborg, Denmark;; † National Centre for Antimicrobials and Infection Control, Copenhagen, Denmark

**Keywords:** letter, animals, drug resistance, fluoroquinolones, microbial, domestic, cephalosporins

**To the Editor:** During the last decade, use of antimicrobial drugs for growth promotion and therapeutic treatment in food animals has received much attention. The reservoir of resistant bacteria in food animals implies a potential risk for transfer of resistant bacteria, or resistance genes, from food animals to humans. Subsequent emergence of infections in humans, caused by resistant bacteria originating from the animal reservoir, is of great concern. These unintended consequences of antimicrobial drug use in animals led to termination of antimicrobial growth promoters in food animals in countries in the European Union, including Denmark, where the consumption of antimicrobial drugs by production animals was reduced by 50% from 1994 to 2003 (*1*).

In Denmark, the VetStat program monitors all veterinary use of medicines for animals. VetStat is based on reporting from the pharmacies and from veterinary practitioners and contains detailed information, such as animal species, reason for prescription, and dosage on each prescription. In Denmark, antimicrobial drugs can be obtained only by prescription and only at pharmacies.

So far, use of antimicrobial drugs in companion animals has received little attention; monitoring programs have focused on antimicrobial drug consumption in food animals. According to data generated by the VetStat program in 2003, consumption of fluoroquinolones and cephalosporins in companion animals was substantial when compared to consumption in food animals (*1*). Fluoroquinolones and cephalosporins are antimicrobial drugs ranked by the U.S. Food and Drug Administration as critically important in human medicine, and for which emergence of resistant bacteria is especially undesirable (*2*). Considering the shared environment of humans and companion animals, transfer of resistant bacteria or mobile resistance determinants from companion animals to humans would be possible, and emergence of resistance to fluoroquinolones and cephalosporins in companion animals should be a matter of concern.

Several scientific publications have reported the occurrence of the same resistance genes in companion animals and in humans (*3**–**6*) and the possible transfer of bacteria between companion animals and humans (*3**–**9*). Companion animal owners and their families are likely in close contact with their animals daily, which provides the opportunity for transfer of bacteria between companion animals and humans. A large proportion of the human population presumably has daily contact with companion animals, not only in Denmark but also in other countries. In Denmark, 20% of families own dogs and 16% own cats (*10*).

In 2002, legal restrictions aimed to reduce the usage of fluoroquinolones in food animals were imposed in Denmark. The total annual consumption of fluoroquinolones in animals (companion and food animals) in Denmark was reduced from 183 kg in 2001 to 53 kg in 2003 (*1*). Of these 53 kg of fluoroquinolones, almost half (24 kg) was used in companion animals (data based on reporting on use in veterinary practice and sales from pharmacies on prescription). These data document that fluoroquinolones remain widely used for infections in companion animals, even though the emergence of fluoroquinolone resistance in bacteria is especially undesirable and regarded as a human health hazard. A similar situation exists with cephalosporins. The total consumption of cephalosporins in animals (companion and food animals) in Denmark in 2003 was 461 kg, of which more than half (254 kg) was consumed by companion animals (*1*).

Thus, a comparatively small number of companion animals (550,000 dogs and 650,000 cats) (*10*) consume approximately the same amount of fluoroquinolones and cephalosporins as consumed annually in the much larger population of food animals in Denmark (23 million slaughter pigs, 130 million broiler chickens, and 1.2 million cattle and dairy cows) (*10*). We do not believe that antimicrobial drugs are more generously prescribed for companion animals in Denmark than in other industrialized countries. Rather, the data presented here reflect the apparent contrast between policies of antimicrobial drug use for food animals and policies for companion animals. The use of these antimicrobial drugs is avoided or restricted in food animals to minimize spread of resistance, while in companion animals prescription continues unimpeded. This situation may create undesirable antimicrobial drug resistance in bacteria, which may subsequently spread to humans from the previously neglected reservoir in companion animals.
